# Mechanochemical Modeling of Dynamic Microtubule Growth Involving Sheet-to-Tube Transition

**DOI:** 10.1371/journal.pone.0029049

**Published:** 2011-12-20

**Authors:** Xiang-Ying Ji, Xi-Qiao Feng

**Affiliations:** Institute of Biomechanics and Medical Engineering, Department of Engineering Mechanics, Tsinghua University, Beijing, China; University of Akron, United States of America

## Abstract

Microtubule dynamics is largely influenced by nucleotide hydrolysis and the resultant tubulin configuration changes. The GTP cap model has been proposed to interpret the stabilizing mechanisms of microtubule growth from the view of hydrolysis effects. Besides, the growth of a microtubule involves the closure of a curved sheet at its growing end. The curvature conversion from the longitudinal direction to the circumferential direction also helps to stabilize the successive growth, and the curved sheet is referred to as the conformational cap. However, there still lacks theoretical investigation on the mechanical–chemical coupling growth process of microtubules. In this paper, we study the growth mechanisms of microtubules by using a coarse-grained molecular method. First, the closure process involving a sheet-to-tube transition is simulated. The results verify the stabilizing effect of the sheet structure and predict that the minimum conformational cap length that can stabilize the growth is two dimers. Then, we show that the conformational cap and the GTP cap can function independently and harmoniously, signifying the pivotal role of mechanical factors. Furthermore, based on our theoretical results, we describe a Tetris-like growth style of microtubules: the stochastic tubulin assembly is regulated by energy and harmonized with the seam zipping such that the sheet keeps a practically constant length during growth.

## Introduction

As the most rigid cytoskeletal element in cells, microtubules have a well-marked lattice structure, consisting of regularly arranged *α*- and *β*-tubulin heterodimers (e.g., [1], [2]). The dimers bind head-to-tail along their longitudinal direction into polar protofilaments, which, in turn, associate laterally in a staggered manner, rendering an elegant tubular and chiral structure. In spite of their mechanical firmness and lattice regularity, the dynamic and evolutionary attributes are intrinsic and essential for microtubules to fulfill their various significant functions in cell divisions and other intracellular biological processes (e.g., [2]–[4]).

Microtubules suffer stochastic transitions between growing and shrinking. Such dynamic processes are highly coupled with the hydrolysis of nucleotides bound on the assembled tubulins [5], [6]. Dimers in different nucleotide states assume different curvatures [7], and two distinct protofilament configurations, curved or straight, are resulted in, depending on whether GDP or GTP is bound [8], [9]. The intrinsic bending characteristic of GDP-tubulins is incompatible with the canonical microtubule lattice [10]. To elucidate the physical mechanisms by which a microtubule composed mainly of bending GDP-tubulins can still keep stable growth, the GTP cap model has been proposed [11], [12]. It hypothesizes that only when the rate of tubulin assembly exceeds the rate of GTP hydrolysis, can some newly-added GTP-tubulin layers maintain as a cap at the growing end of the microtubule. The GTP cap can sustain the uniform lattice and the continuous growth of the microtubule, and its disappearance will cause the depolymerization. Despite the logical elegance of this cap model, yet there is a shortage of sufficient evidence for the existence of the GTP cap, and disputes exist about its size [13], [14] and the inside GTP distributions [15], [16].

Experimental observations also reveal that a microtubule end can assume far more colorful conformations than mere straight or curved [17]. Typically, an open and outward-curved sheet is imaged at the growing end by cryoelectron microscopy (cryo-EM) [18], [19]. Such a sheet structure has been considered an interphase during microtubule growth [20]–[22]. The growth is achieved by the sheet closure which involves a distinct transition of curvature, from longitudinal to lateral. The feasibility of this growth pathway has been experimentally validated by Wang and Nogales [10]. They showed that at low temperature, tubulins binding GMPCPP, a non-hydrolysable GTP analogue, can form ribbons, in which the protofilaments have a radial bend of about 5° between two adjacent dimers. As temperature rises, the ribbons directly convert into tubes. It is thus suggested that the GMPCPP ribbons structurally correspond to the curved sheets at the growing microtubule end [10]. The sheet-to-tube growth mechanism can interpret, quite successfully, such phenomena observed in microtubule growth as the formation of a seam. The seam, apparently being a linear lattice defect, may virtually offer important binding sites for associated proteins to help zip the microtubule [23].

Prominently, the above-described growth mode, which involves a conversion of end conformations, itself provides a stabilizing mechanism for microtubule growth [9], [24]. Due to this mechanism, which is referred to as the conformational cap model, the sheet is more stable than the zipped microtubule body [25]. The sheet closure happens stochastically [9], and its complete closure into a blunt-ended tube would induce microtubule depolymerization [26]. Despite this well-conceived essentiality of the sheet structure in microtubule growth, the detailed mechanisms remain vague.

Though the conformational cap and the GTP cap models offer two different stabilizing mechanisms, a microtubule does not face an either-or choice. Actually, the conformational cap does not contradict the GTP cap but provides further guarantee for a stable microtubule growth. Also, the conformational change contributes bonus energy accumulated in microtubule lattice in addition to the energy from GTP hydrolysis [27], [28], i.e., the elastic strain energy caused by the curvature transition [25]. However, the detailed correlation between these two caps and the relation between closure and hydrolysis remain unclear. Structure analysis reveals that only GTP-tubulins can form lateral contacts compatible with the microtubule lattice, and the hydrolysis is not necessary for sheet closure and microtubule growth [10]. However, this conclusion does not dictate a direct link between closure and hydrolysis, but only suggests that hydrolysis could happen after closure. Further investigation is desired to determine whether closure triggers hydrolysis [18], [29]. On the other hand, if a sheet composed of only GDP-tubulins can stabilize the growth phase [25], [30], the essential role of pure mechanical factors in cell physiology is signified since the conformational cap works mainly mechanically.

These previous experimental findings clearly demonstrate the intrinsic and strong coupling between mechanical and biochemical mechanisms, which is prevalent and vital for the dynamic behavior of microtubules. A deeper understanding of the appealing microtubule characters calls for well-defined theoretical models. Much effort has already been directed towards the modeling of microtubule dynamics. The switch between growing and shrinking and the corresponding length variation have been described by differential equations (e.g., [13], [31], [32]). A detailed probe of the evolutions of conformation and energy inevitably needs finer three-dimensional simulations. Some examples have been given by VanBuren et al. [33] and Molodtsov et al. [34], who successfully accounted for the strain energy changes induced by the association and disassociation of individual dimers. As yet, however, there is still a lack of theoretical investigation on the dynamics of the sheet-to-tube growth mode. The spatial energy distribution and variation, as well as the complete growth process, under mechanical–chemical coupling regulations have rarely been addressed.

Recently, we established a coarse-grained model for studying the macroscopic behavior of microtubules [35]. The equilibrium conformation of a growing, sheet-ended microtubule, in which protofilaments are both bending and twisting, is obtained via a form-finding process based on the defined interactions between monomers. As an illustration of this model, a radial indentation process of a microtubule with large deformation has been simulated and the results well fit with the relevant experiments, demonstrating the efficacy of this model in representing a dynamic process involving structure and energy evolutions [35]. It has also been demonstrated that the sheet structure is energetically stable. In this paper, we will employ this model to investigate the microtubule growth process, which involves a sheet-to-tube transition and shows a mechanical–chemical coupling. We comprehensively calculate the potential energy by taking into account the intrinsic curvatures of both GTP- and GDP-tubulins. Such dynamic events as subunit association are treated as stochastic processes regulated by the coupled changes of chemical association energy and mechanical potential energy. The influence of GTP hydrolysis on the intrinsic conformation of tubulins is also taken into account. This study provides insight into the conformational cap hypothesis and the stabilizing mechanisms in the microtubule growth process.

## Methods

### Coarse-grained model of microtubules

**Figure 1:**
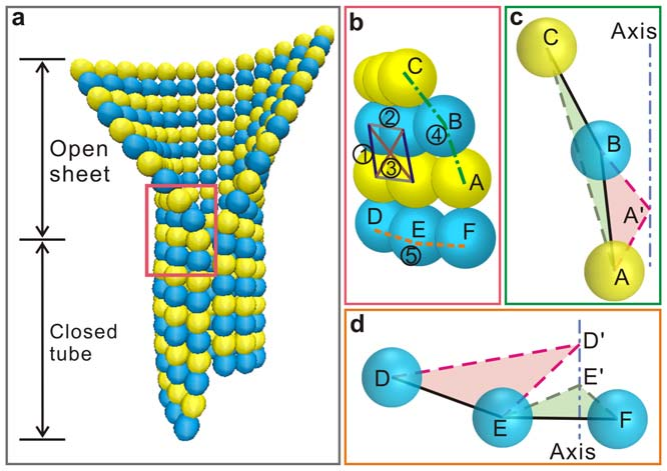
Model of a sheet-ended microtubule. (a) The equilibrium structure of a sheet-ended microtubule revealed in Ref. [35]. (b) The partial enlarged view of the microtubule structure marked in the red box in (a), showing (1) the longitudinal tension or compression interaction, (2) the lateral tension or compression interaction, (3) the diagonal tension or compression interaction, (4) the longitudinal bending interaction, and (5) the lateral bending interaction. (c) The longitudinal bending and the dihedral bending interactions. The bending interaction considers the supplementary angle of the angle A–B–C, and the dihedral angle is defined between the plane A–B–C and A–A′–B. (d) The lateral bending and dihedral bending interactions. The bending interaction considers the supplementary angle of the angle D–E–F, and the dihedral angle is defined between the plane D–D′–E and E–E′–F.

In our previous work [35], a model has been developed to simulate the dynamic behavior of microtubules by considering their structural complexity and mechanical–chemical coupling features. A standard microtubule with 13 protofilaments and a pitch of 3 monomers is considered (Fig. 1a). Due to its structural chirality, the microtubule has a seam, where the laterally attached tubulins are *α–β*, rather than the prevalent contacts of *α–α* and *β–β* in the otherwise positions. Differing from the preexisting coarse-grained models [33], [34], the extending sheet structure is spotlighted in this model. The specific equilibrium sheet-ended microtubule conformation is determined via a form-finding process by minimizing the interaction potential of the whole microtubule. Three major types of interactions among the adjacent monomers in the model are the tension or compression interactions regarding their distance variations, the bending interactions regarding their angle variations, and the dihedral bending interactions regarding their dihedral angle variations, as shown in Fig. 1.

For the tension or compression interactions, the distance variations between two neighboring monomers along the longitudinal, lateral, and diagonal directions are taken into account (Nos. 1, 2, and 3 in Fig. 1b). The equilibrium longitudinal and lateral intervals are set as 4.0 nm and 5.2 nm, respectively, according to the canonical microtubule lattice [29]. Numerical simulations show that the diagonal interactions [36] scarcely influence the calculation results but are essential for the quick convergence of the simulation [35]. Due to the chirality of the microtubule, the lattice is oblique and two equilibrium diagonal intervals are calculated as 5.9988 nm and 7.1242 nm.

The bending interactions correspond to the elastic energy induced by the angle variations made by three adjacent monomers. The longitudinal and lateral bending interactions are numbered as 4 and 5 in Fig. 1b, respectively. A longitudinal curvature is intrinsic for the structure of protofilaments and the bending becomes even more distinct when the bound GTP is hydrolyzed. Protofilaments of GDP-tubulins are highly curved and normally peel off into a horn-like shape when the microtubule experiences depolymerization [37]–[39]. Following the structure analysis by Nogales and Wang [40], the longitudinal equilibrium angle between two neighboring monomers is assumed to be 5° for GTP-tubulins and 18° for GDP-tubulins. As to the bending along the lateral direction, in view of the commonly observed two-dimensional sheets of tubulin assemblies, which are the precursors of microtubule formation [19], [22], [25], [40] or induced in presence of some cations [41], [42], the equilibrium angle is assumed to be zero but its exact value is yet unknown.

The longitudinal and lateral dihedral bending interactions are introduced to depict the coherence of the deformations along the protofilament and the helical turn, respectively. The dihedral interactions describe the energy variation caused mainly by lattice twisting and are described by the relative angular change of an assigned molecular plane with respect to its canonical plane [35]. For three adjacent monomers along a same twisted protofilament (labeled as A, B, and C in Fig. 1c), the relative twisting can be characterized with reference to the locations of the monomers A and B, with A′–A being the radial directions. The longitudinal dihedral angle is thus defined as the angle between the plane formed by the three monomers, A–B–C, and the reference plane, A′–A–B. For three adjacent monomers along a same helical turn (labeled as D, E, and F in Fig. 1d), D′–D and E′–E are the radial directions. The planes D′–D–E and E′–E–F do not coincide when the helical turn is twisted and are used to define the lateral dihedral angle. Therefore, both the equilibrium longitudinal and lateral dihedral angles are set to be zero.

For all the seven interactions, the following form of potential functions is assumed
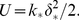
(1)For the longitudinal, lateral and diagonal distance variations, the longitudinal and lateral angle variations, and the longitudinal and lateral dihedral angle variations, the parameters 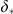 in Eq. (1) stands for 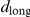, 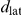, 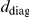, 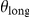, 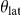, 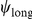, and 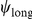, respectively; and the corresponding elastic coefficients are 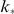 = 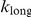, 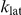, 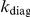, 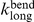, 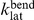, 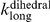, and 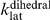, respectively. The values of the constants 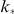 are determined as follows. 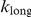 and 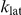 are determined following the results of atomistic dynamic simulations and mesoscale models in the literature [43], [44]. 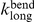 and 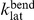 are determined by treating the tubulin assembly with the continuum mechanics method [35]. 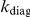, 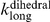 and 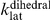 are assumed empirically and their values have little influence on the calculation results. These parameter values have been validated through a series of numerical simulations in our previous paper [35] and will be further testified in this study. The definitions of interactions and the interaction constants are summarized in Table 1. For more details, please refer to Ref. [35].

**Table 1:** Interaction definitions in the model.

	Interaction	Interaction potential	Interaction constant	Value
1	Longitudinal tension or compression	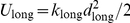	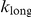	3.0 nN/nm
2	Lateral tension or compression	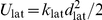	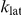	14.0 nN/nm
3	Diagonal tension or compression	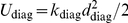	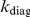	3.0 nN/nm
4	Longitudinal bending	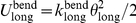	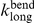	2.0 nN·nm
5	Lateral bending	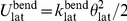	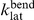	8.5 nN·nm
6	Longitudinal dihedral bending	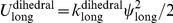	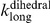	0.04 nN·nm
7	Lateral dihedral bending	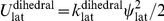	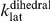	0.17 nN·nm

We emphasize here that the defined interactions form an integrated description of the microtubule conformational and mechanical properties. The rationality and robustness of the coarse-grained and mechanical–chemical coupling model have been demonstrated by a series of simulations on the equilibrium conformations of sheet-ended microtubules and the structure evolution under radial indentation [35].

### Free energy of association

For polymerizing polymers, the free energy of association can be divided into two additive parts [36], [45]. One is beneficial for association, including the free energy associated with the interfaces or bonds between subunits, called “bond energy”. Let 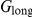 and 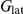 denote the bond energies for longitudinal and lateral associations, respectively. The other part is unfavorable for association, denoted by 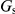. It is the free energy required to immobilize a subunit in the polymer, to which the most important contribution is the loss of entropy due to association. Electrostatic, hydrophobic, and other noncovalent interactions are a bit loose rather than rigid. Therefore, the subunits are not totally fixed but have some freedoms to rotate and vibrate. Evidently, the loss of entropy in this case is less than that when the translational and rotational degrees of freedom are completely lost [46], [47]. Here, we take 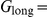
−19 k_B_T/dimer, 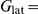
−4 k_B_T/dimer, and 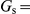
11 k_B_T/dimer. These values match the theoretical results of chemical reaction kinetics [36] and are verified by computer simulations [48].

**Figure 2:**
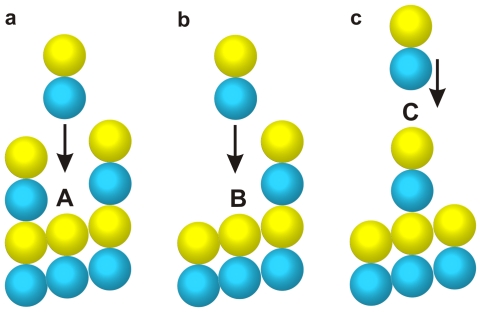
Sites for a coming tubulin dimer to assemble: (a) the dimer inserting into a gap, (b) the dimer associating a single-sided neighbor, and (c) the dimer falling upon the crest.

Fig. 2 shows the free association energies for the assembly of a tubulin dimer at different sites. In the first case, a dimer filling into the gap between two long protofilaments (Fig. 2a) will form two lateral bonds and a longitudinal bond with the pre-assembled subunits and, therefore, the corresponding association energy is calculated by
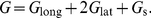
(2)For the second case, a dimer associating at the side of a long protofilament (Fig. 2b) gets a lateral bond and a longitudinal bond. Correspondingly, the association energy equals
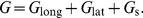
(3)In the third case, only a longitudinal bond can be formed when a dimer assembles at the crest of the microtubule (Fig. 2c) and the association energy is
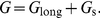
(4)The competition and balance of the association energy and the potential energy will dictate the assembly process.

### Basic algorithm

According to the conformational cap model, the growth process of a microtubule involves the addition of tubulin dimers at the protofilament tips and the closure of sheet, both of which exert conformational perturbations on the microtubule structure. The simulation is performed using an equilibrium sheet-ended microtubule conformation [35] as a starting point. When a conformational alteration happens, the following iteration steps are repeated until the total interaction energy of the entire microtubule converges:

- The potential energies of all monomers are computed following the above-defined interactions.
- The force exerted on each monomer is determined from the derivative of the potential energy with respect to its coordinates.
- The Verlet integration method is used to obtain the new positions of all monomers. In this model, the units of lengths, forces, and masses are nm, nN, and kDa, respectively. The time step is taken as 0.1, which corresponds to 1.29 Ps in real time scale. Each tubulin monomer has the mass of 55 kDa [49], [50]. The calculated velocities of all monomers are rescaled to keep a constant average kinetic energy.
- All monomer positions are updated, and then a new iteration cycle starts from Step (i). If the difference between the root mean square values of the total potential energy given by the last ten and twenty steps is smaller than 0.0001, we judge that the calculation has been converged and then terminate the iteration.

When the equilibrium state has been achieved, a new conformational disturbance is allowed to occur such that the dynamic growing process of a microtubule can be simulated.

### Integrated thermodynamic description of microtubule growth

The attention of this study is focused on the microtubule growth, which involves the sheet-to-tube transition and the tubulin assembly. The assembly process is treated by a stochastic thermodynamic description, in which tubulin dimers are randomly assembled at the growing sheet end. Following the interaction potential described above, the equilibrium potential energy of the whole microtubule after the assembly is calculated and denoted as 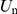, where the subscript *n*, taking a value from 1 to 13, stands for the protofilament on which the assembly happens. Let 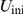 denote the equilibrium potential energy before this assembly happens, and 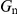 the free energy of association regarding this assembly at the *n*-th protofilament. Then, the energy difference caused by a potential assembly with respect to the initial energy is
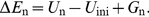
(5)If 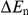 is positive, the corresponding assembly will not happen. If 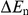 is negative, the assembly can happen with a particular probability 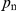:
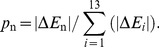
(6)After the assembly, the equilibrium conformation is calculated. Thereafter, a new computation step begins and new tubulin dimers add onto the microtubule tip.

To date, no direct experimental observation has been reported on the closure process of microtubules at the molecular scale. Based on the considerations of the lateral interaction between monomers, we characterize the closure as a monomer-based process, in which the seam is zipped by the consecutive linking of monomer pairs. The closure of the monomer pair at the opposite edges of the sheet bottom is realized by taking into account the additional lateral tension or compression, bending, and dihedral bending interactions relevant to the monomer pair. Thus, the whole microtubule conformation will evolve into a new equilibrium state.

To date, there still exists ambiguity in the literature for the GTP hydrolysis process, especially for the relationship between sheet closure and GTP hydrolysis [10], [18], [25], [29], and the distribution of GTP-tubulins in the microtubule [16], [51]–[53], adding difficulties to the modeling. In this paper, we will not examine the molecular mechanisms of GTP hydrolysis. The GTP hydrolysis is not included in the thermodynamic process of microtubule polymerization but, instead, is treated as a forced event. The GTP hydrolysis alters the intrinsic curvature of tubulins and thus the equilibrium longitudinal bending angle. Unless otherwise specified, we will use the following rule of hydrolysis. The sheet is assumed to be consisted of GTP-tubulins whereas the closed part is totally hydrolyzed. In other words, the whole helical turn at the root of sheet will hydrolyze synchronously with its closure [26]. This relation is irrespective of the causality of GTP hydrolysis and sheet closure. Different hydrolysis rules can be easily implemented into the simulations. To illustrate the effect of hydrolysis on the sheet-to-tube transition, an example based on a random hydrolysis rule and different GTP distributions in the sheet will also be provided in the following subsection.

## Results

### The sheet-to-tube transition process contributes stored energy to the microtubule lattice

**Figure 3:**
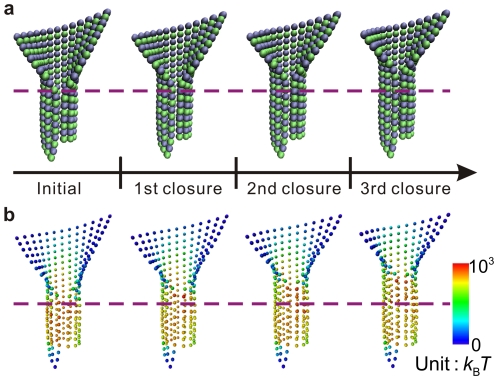
Snapshots of the conformation and potential energy evolutions during a sheet-to-tube transition process. The equilibrium states before assembly and after three consecutive closures are shown: (a) the evolution of the microtubule structure, and (b) the corresponding evolution of the total potential energy.

**Figure 4:**
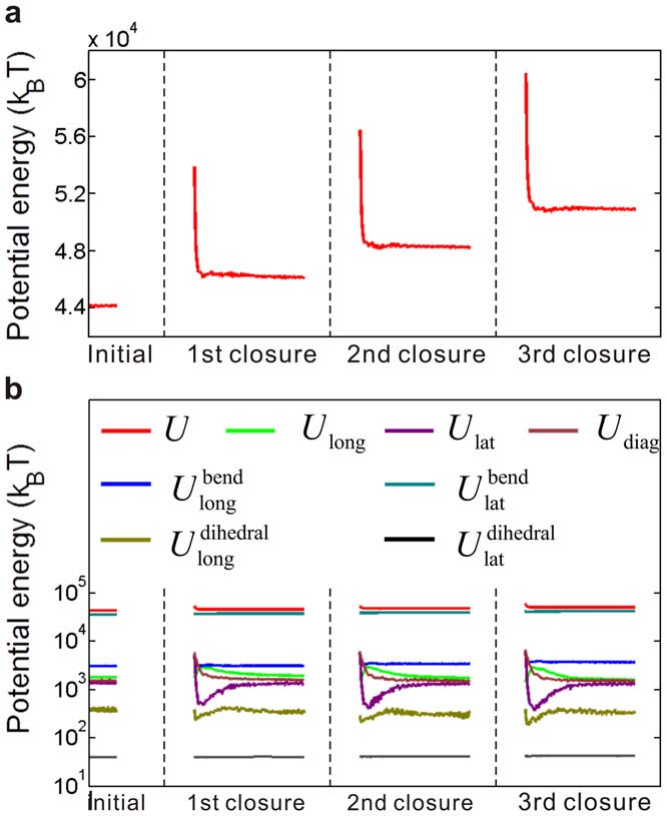
Potential energy evolution during the sheet-to-tube transition process. A continuous zipping of the seam counting three pairs of monomers is characterized. The consecutive closure happens when the microtubule is in an equilibrium conformation. (a) The evolution of the total potential energy, from which the energy barrier and energy difference between two equilibrium states are clearly detected. (b) The evolution of the seven energy components. Respecting the differences of orders of magnitude, a semilogarithmic coordinate is adopted.

It is widely accepted that the hydrolysis of GTP bound on the added dimers helps the accumulation of energy constrained in the microtubule lattice. In addition, the conformational cap model proposes that the curvature transition during the sheet-to-tube transition would also contribute to the lattice energy [25]. We simulate the closure process of a microtubule and analyze the variation of the potential energy by using the presented model. The original sheet structure is composed of GTP-tubulins and measures ten monomers in length. Once a monomer pair closes, the GTPs bound on tubulins of the newly closed helical turn will hydrolyze, corresponding to a change of their equilibrium longitudinal bending angles from 5° to 18°. Fig. 3 shows some snapshots during the continuous sheet-to-tube transition process. The equilibrium conformations at the beginning and after the successive closure of three monomer pairs are visualized by the package VMD [54], as shown in Fig. 3a. The corresponding distributions of the total potential energy at the four states are visualized by OVITO [55], as shown in Fig. 3b. The complete transition process of the entire sheet and the energy changes are given in Movie S1 in the Supporting Material. Fig. 4 shows the evolution of the total potential energy and the seven components of interaction energy during the successive closure process of three monomer pairs. It is demonstrated that the closure is steadily propelled. Both the energy barrier and the energy difference between two subsequent equilibrium states are identical during the whole closure process. The energy barrier is calculated to be around 10^4^ *k*_B_ *T*, which is the value of energy needed for a single monomer pair to close. When a monomer pair has been zipped and the whole microtubule has become stable, the accumulated energy (the energy stepping in Fig. 4) amounts to about 2400 *k*_B_ *T*, approximately 10 times higher than the energy from hydrolysis [29], [56].

It is noted that after each closure of a monomer pair, the potential energy elevates. Among the seven parts of potential energy, the lateral bending potential energy makes the most significant contribution to the total energy. Our previous work about the equilibrium conformation of a sheet-ended microtubule provided the concrete distributions of the seven potential energy components and demonstrated their rationality from a bio-physiology perspective. Here, we further demonstrate that the sheet-to-tube transition calls for energy input. The conformational cap hypothesis states that only after the sheet entirely closes, can depolymerization happen from the blunt microtubule end [26]. Therefore, it is conclusive that the energy-required sheet-to-tube transition is an effective mechanism for ensuring the stable growth of microtubules and preventing depolymerization. In addition, the sheet-to-tube process contributes greatly to the energy accumulation in the microtubule lattice, and the stored energy will be released to do work during the later microtubule depolymerization [57].

The binding condition along the seam is crucial but has not been clearly revealed in the literature. It has also been recognized that some other proteins (e.g., EB1) are involved in the zipping process and contribute energy in the closing process of the sheet [22], [23], [58], [59]. Without considering this complexity, we take the occurring of closure as a hypothesized fact. For this reason, the potential energy is unfavorable for the closure process and some free energies of association and other promoting interactions are provided by some mechanisms associated with other proteins. The tubulins along the lateral edges of the sheet may have a high free energy of association to complete the sheet-to-tube transition, or some microtubule-associated proteins such as EB1 can help the zipping of the seam, which will be discussed further in the Discussion Section.

### The intrinsic curvature of GTP-tubulins scarcely affects the function of conformational cap

**Figure 5:**
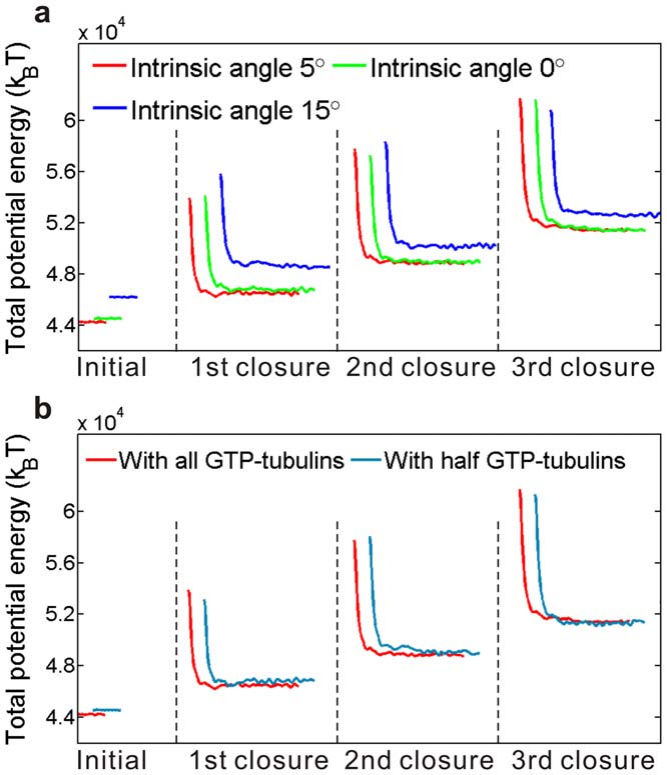
Comparison of the energy barriers and energy differences during sheet-to-tube transitions under different sheet structures: (a) the intrinsic curvatures of GTP-tubulins are of three different values, and (b) the sheets are in two different nucleotide states. In both panels, the red lines represent the result for the standard model shown in Fig. 4. For clarity, the three sets of data have been offset horizontally, but not vertically.

Though straighter than GDP-tubulins [60], GTP-tubulins are not perfectly straight as widely assumed [61]. A slight bend of about 5° was exhibited at the intra-dimer interface of each GTP-tubulin [40]. Using the defined model, we now explore the influence of this intrinsic curvature of GTP-tubulins on the growth process of microtubules. The energy variations during the closure of GTP sheets with an intrinsic curvature of 5° (standard), 0° and 15° are compared in Fig. 5a. It is seen that these three cases have no distinct difference either in the activation energy or the equilibrium energy stepping. This means that neither the conformation nor the energy evolution during the sheet-to-tube transition is sensitive to the longitudinal curvature. The intrinsic curvature should be dictated mainly by some structural factors and seems to be not critical to the growth process and stabilizing mechanisms of microtubules. This result supports the recent investigation about the intrinsic bending of microtubule protofilaments by Grafmüller and Voth [62], who, through large-scale atomistic simulations, concluded that no observable difference exists between the mesoscopic properties of intra-dimers and inter-dimers. The distinct curvature difference between polymerizing and depolymerizing protofilaments may majorly due to their lattice constraint.

### The conformational cap works independently of the nucleoside states

The relationship between the GTP cap and the conformational cap has long been speculated but remains unclear [10], [25]. In this subsection, we will test whether the conformational change caused by GTP hydrolysis will resist the sheet-to-tube process and thus inhibit the microtubule growth.

Firstly, we compare two microtubule models with a GTP cap of different lengths, in which the locations of GTP-tubulins are assumed orderly, that is, there exist some layers completely constituted of GTP-tubulins, capping the microtubule. In the first model, the sheet is composed only of GTP-tubulins and the closed part is totally hydrolyzed. In the second model, half of the sheet is hydrolyzed and, more specifically, we assume that the upper part of the sheet is composed of GTP-tubulins whereas the lower part is composed of GDP-tubulins. The GTP hydrolysis of the lowest GTP-helical turn is treated as a process synchronous with the closure of the tubulin pair at the root of the sheet. Fig. 5b compares the energy evolutions during the closure process in the two microtubule models. For the sheets with different GTP distributions, both the values of the activation energies and equilibrium energy differences are similar, indicating that the conformational cap could function independently of the GTP cap.

**Figure 6:**
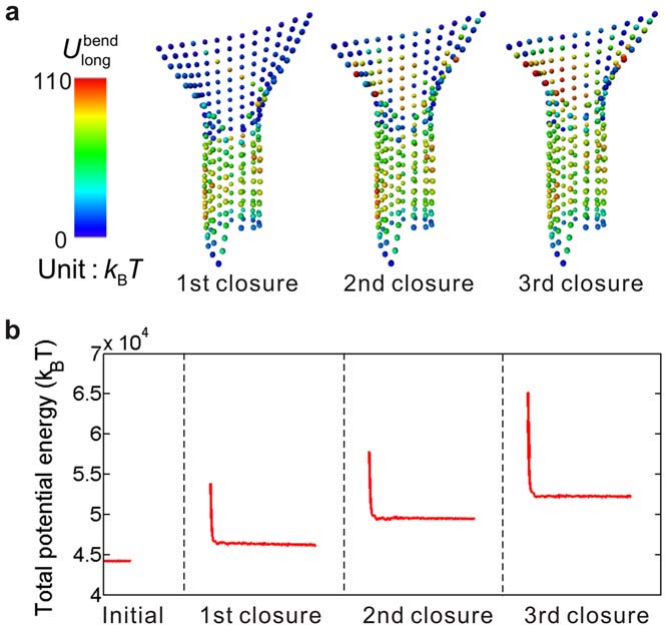
Potential energy evolution during a sheet-to-tube transition with randomly distributed GTPs. (a) Snapshots of the longitudinal bending potential distribution during a time span of three closures of a monomer pair. The illustrated states are in equilibrium. (b) Evolution of the total potential energy.

Secondly, we investigate the influences of random GTP distribution and hydrolysis. An irregular and varying distribution of GTP-tubulins is the case suggested by many experiments [15], [16], [51], [52]. A microtubule with a sheet of interlaced GTP-tubulins and GDP-tubulins is considered. At each simulation step of the sheet closure, the hydrolysis of GTPs bound on tubulins in the sheet happens randomly. As an example, we consider a sheet with the following initial composition of tubulins. All the tubulins in the sheet are GTP except that the *n*th tubulin counted from the bottom of the sheet in the *n*th (*n* = 1–10) protofilament and the *n*th and (*n*+1)th tubulins in the *n*th (*n* = 11–13) protofilament have been hydrolyzed as GDP. The closed part of the microtubule is assumed to be purely composed of GDP-tubulins. Since the sheet-to-tube transition has little relevance with the closed part, its nucleoside state is indifferent. The following hydrolysis rule is assumed in this example. For each closure, a random number ranging from 0 to 1 is generated for each GTP-tubulin in the sheet, and if the number exceeds 0.5 the GTP will hydrolyze. The entire sheet-to-tube transition under these assumptions is well simulated. Fig. 6a shows the snapshots of the longitudinal bending energy distribution during the successive closure process of three monomer pairs. Here, the protofilaments labeled from 1 to 13 are counted counterclockwise round the tube from the right side of the seam. At the beginning, a spiral line of higher energy value is formed due to our assumption about the initial state of the nucleoside distribution. Though the local intrinsic curvature may change violently due to the GTP hydrolysis, a smooth sheet structure is maintained. In the following steps of closure, the random hydrolysis leads to a disorder distribution of longitudinal bending interaction energy. Fig. 6b shows the evolution of the total potential energy, which involves an evident energy barrier and energy stepping. It is demonstrated that the spatiotemporal stochastic curvature variations caused by GTP hydrolysis will not interfere with the stabilizing role of the sheet structure in microtubule growth.

Wang and Nogales [10] have stated that the bending of GDP-tubulins is incompatible with the formation of canonical lateral interaction in microtubules. Here, our modeling is based on the premise that the lateral interactions have been pre-defined except those along the seam. Though insufficient to completely testify the relation between the longitudinal bending conformation and the lateral interaction, our results demonstrate that the curvature transition and the sheet closure can be achieved by highly curved GDP-tubulins. This indicates that the function of the conformational cap could be decoupled from the GTP cap. Despite the likelihood that the substructure change in hydrolyzed tubulins would resist the formation of lateral interactions, at least from the view point of energy mechanism, hydrolysis is allowed to the tubulins on the sheet before closure and the microtubule growth will not be interfered by the nucleotide state. In addition, our simulations evidence the stabilizing role of the conformational mechanism in microtubule growth, and highlight the significance of mechanical factors in microtubule behaviors.

### A conformational cap should include at least two tubulin dimer layers

**Figure 7:**
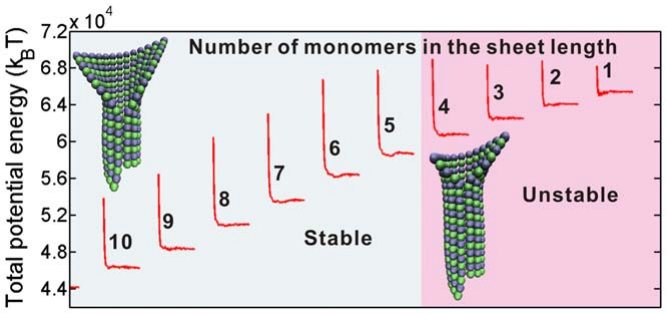
Energy barriers and energy steppings induced by a monomer pair closure for ten sheets of different lengths, varying from 1 to 10 monomers in the longitudinal direction. The ten microtubule models are composed of protofilaments of the same length. The total potential energies are at different levels since the length of closed parts of the ten models are different.

The cap length has long been a controversial issue [16], [63], [64]. In this subsection, we will investigate how the energy barrier and the equilibrium energy stepping during the zipping of a monomer pair depend on the sheet length. For ten sheets of different lengths from 1 to 10 monomers, the numerical results of the potential energy evolution induced by the closure of a monomer pair are compared in Fig. 7. Clearly, the energy barrier and the energy stepping for the sheets containing 4 to 10 monomers in each protofilament along the length direction (the left part of Fig. 7) are almost identical, but they are much higher than those in shorter sheets (the right part in Fig. 7). If the sheet is shorter than the length of two dimers, the activation energy to close the sheet drops distinctly and thus the whole open sheet would experience a swift and unstable transition into a tube. In this case, the sheet structure will lose its stabilizing effect and then depolymerization will occur.

Some recent experimental and theoretical works about the GTP cap's size concluded that an effective GTP cap should include at least two dimer layers [15], [34]. The minimum size of the conformational cap estimated by our model is in accordance with that for the GTP cap reported in the previous studies. Such a consonance hints the potential direct relevance between closure and hydrolysis. As estimated by some researchers [29], hydrolysis can be catalyzed by the closure due to the induced energy accumulation.

### Microtubule growth likes Tetris

We have demonstrated that the open sheet structure at the growing end can stabilize the microtubule growth and have testified the sheet-to-tube growth style. However, if the closure speed is faster than the tubulin assembly rate at the tip, the sheet structure will be fully closed into a blunt microtubule end and, thereafter, depolymerization may be triggered [26]. Therefore, a stable growth should rest on the harmonization between closure and polymerization, which keeps the sheet having a practically constant length of at least two dimer layers. In this case, the seam's zipping will experience a self-similar propagation, which conjures up the reverse process of a stable crack advancement.

**Figure 8:**
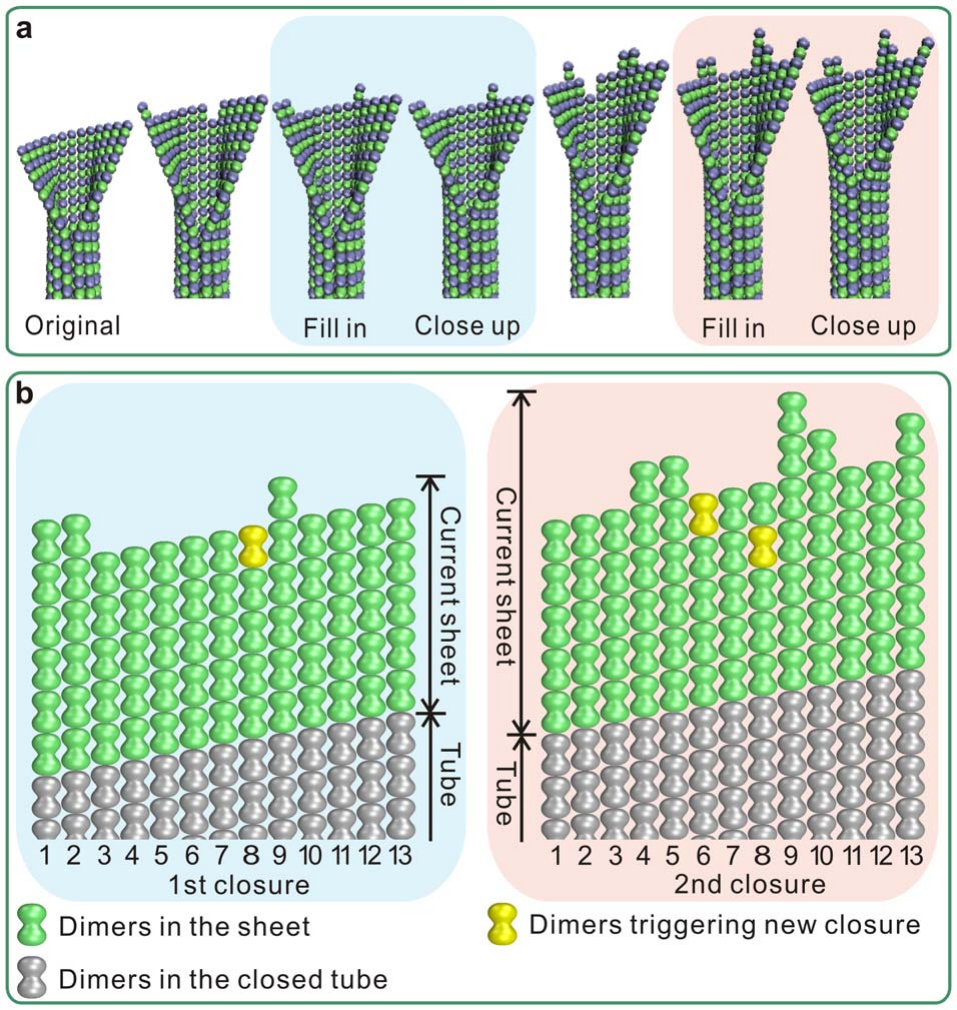
Harmonized assembly and closure during microtubule growth. (a) Snapshots of the sheet structure during a time span of two subsequent closures of a monomer pair. When a layer at the end of the sheet has been fully filled by newly added tubulins, the seam will be zipped a same length. The blue and red-highlighted structures show two processes of the assembled dimers fulfilling a complete layer and the subsequent seam zipping up. (b) The microtubule end development at the two closures in (a). The two yellow-colored dimers are those finally fulfill a layer and trigger a corresponding closure.

Thereby, a Tetris-like growing style is extrapolated for the compatibility of assembly and closure during the microtubule growth. Tubulin dimers assemble stochastically at the end of a microtubule under the regulation of energy. When the newly added tubulins have formed a complete layer, the bottom layer at the sheet will transit to close and the seam will advance a dimer-length. Fig. 8 shows some snapshots of the “fill in–close up” process, and the dynamic process is shown in Movie S2. The sheet length is nearly constant so that the microtubule can grow steadily; conversely, if the sheet is quickly closed into tube, the game will soon be over.

## Discussion

### Physiological indications

#### Actual sheet length

The above calculations put forward the hypothesis that the sheet keeps a nearly constant length of at least two dimers during the sheet-to-tube growth. However, the actual sheet length is yet unknown. We speculate that the sheet length may correlate with the nucleation process, and the length determined in the nucleation is sustained in the subsequent growing stage. It has been supposed that the microtubule nucleation template is likely to have a sheet structure composed of laterally associated short protofilaments. The closure of a sheet will lead to a microtubule embryo [19], [21]. Thus the microtubule nucleation and assembly share the same basic mechanism [25]. For a longitudinally curved sheet to transit its curvature and close into tube, an energy barrier needs to be overcome, which should be higher if more tubulins were zipped. The available energy that can be employed to overcome the barrier may determine the nucleated conformation and, in turn, the sheet length. It is inspiring that the sheet-to-tube nucleation pathway can be simulated under the theoretical framework presented in this paper. Our further study will focus on simulating the nucleation process to probe the critical nucleus, the structural templates, and the influence of nucleation on the subsequent growth.

Alternate likelihood is that the sheet length is relevant with the hunt of the microtubule ends for coming tubulins and regulators of assembly such as microtubule-associated proteins (MAPs). As can be seen from the form-finding results of sheet-ended microtubules [35], the sheets with different lengths have different shapes: a shorter sheet has a rounded top edge and its lateral alignment is more compact. The sheet end offers special recognition sites for tubulins to assemble. The inter-protofilament interfaces may accommodate MAPs [40], [65]. Specifically, some plus-end-tracking proteins (+TIPs) bind the microtubule end with a higher affinity than the wall [66], and one of the proposed mechanisms is their recognition of a unique structural feature of the growing end [67], [68]. These effects should be of direct relevance with the spatial structure of the edge line, the lateral gap between protofilaments, and, therefore the sheet length.

#### Indicated roles of some +TIPs such as EB1/Mal3p

Our simulations have clearly revealed that there exists a steady energy barrier during the closure of a sheet, and the sheet-to-tube process needs to be activated. It has been supposed by experiments that a kind of microtubule associated proteins, EB1 in vertebrates and its homolog Mal3p in schizosaccharomyces pombe, can bind the seam. In the presence of such proteins, the microtubule growth is promoted [23]. However, the details of the molecular and energetic mechanisms of EB1/Mal3p in microtubule growth are as yet unclear and beyond the scope of this study.

Besides the function of helping the seam to zip, some +TIPs are speculated to have some other roles in promoting the microtubule growth. Firstly, +TIPs may facilitate the assembly of longer tubulin oligomers in addition to individual tubulin dimers by associating the dimers along its length in solution and adding the whole oligomer into the growing microtubule [69]. Secondly, because of this template effect of +TIPs for oligomer assembly, the dimers are pre-straightened before adding onto the microtubule end, thus the whole microtubule structure is more firm and the closure is facilitated. These influences of assembly units and configurations on the global microtubule growth can also be conveniently investigated by this model.

#### Mechanical influences of GTP hydrolysis

GTP hydrolysis is directly bound up with the microtubule dynamics. Tubulin dimers bind two GTP molecules. GTP at the N-site of *α*-tubulin is non-exchangeable, whereas GTP at the E-site of *β*-tubulin will hydrolyze into GDP after assembly [70]. It is commonly agreed that the main body of a microtubule is made of GDP-tubulins, although the GTP distribution at the sheet structure at the growing end is yet unclear. However, some experiments suggest that the microtubule lattice also contains scattered GTP-tubulin remnants, meaning that the hydrolysis is sometimes incomplete during polymerization [51], [52]. Currently, the moment and condition for GTP to hydrolyze, as well as the influence of GTP hydrolysis on the properties and behavior of microtubules, is little known. In this paper, microtubule models with a sheet in different nucleotide states have been compared. We have also tested the models in which the tubulins in the closed lattice are not in the same nucleotide state and, namely the conformational state. No distinction is found with respect to the sheet-to-tube curvature conversion process, implying that the conformational change of tubulins resulted from GTP hydrolysis hardly interferes with the mechanic requirement of the global conformation evolution of a growing microtubule. The conformational change may mainly influence the depolymerization process. For example, it weakens the lateral interaction and facilitates the ram's horn-like peeling of protofilaments, and the GTP remnants in the lattice could help rescue the microtubule from shortening [69], [70].

### Model discussions

#### The equilibrium value of the lateral bending interaction

The lateral bending potential energy is essential for the simulation of the microtubule behavior which involves the dynamic closure of the open sheet at its growing end. This type of interaction has not been considered in previous coarse-grained models aimed at simulating the assembly and disassembly of tubulins [33], [34]. Meanwhile, there is a shortage of direct experimental evidence for its value. Considering that the sheet structure is highly likely to be crucially involved in the microtubule nucleation [18], [19], [22] and growth [10], [71], [72] and that the sheet structure is commonly formed by tubulins in the presence of zinc ions [41], [42], the equilibrium value of the lateral bending potential energy is taken to be zero [35]. This treatment is acceptable for revealing the growth stabilizing role of the sheet and the energy accumulation mechanism in the microtubule body. Based on this definition, we have successfully found the equilibrium sheet-ended microtubule structure [35] and have modeled the sheet-to-tube transition process, which are consistent with relevant experiments. Our results serve as effective support for the conformational cap model.

#### Influences of the interaction definitions

Our model has three kinds of interactions that are not fully experimentally based, and their values are assumed as 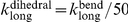, 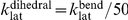, and 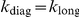 [35]. Our previous work has demonstrated that the variations of the two dihedral angles have little influence on the total energy [35]. The diagonal interaction majorly acts to restrain the fluctuations of tubulin positions and make the calculation converge faster. Here, we examine the influence of these values on the energy evolution during the closure process and validate our assumptions.

**Figure 9:**
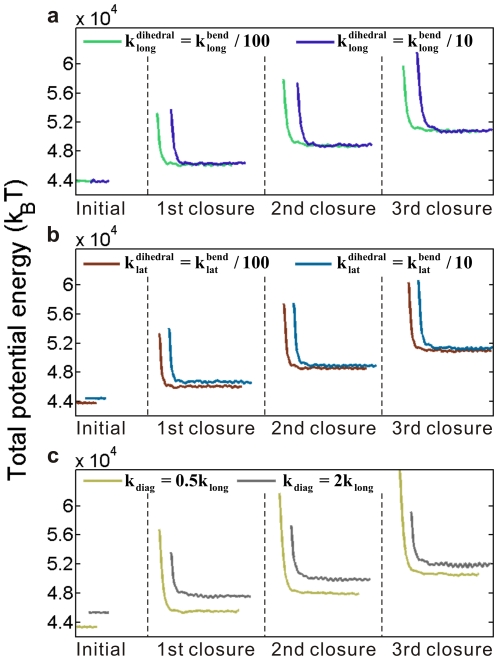
Influences of interaction constants on the energy barrier and energy difference between two equilibrium states during closure. (a–c) Influences of the interaction constants of lateral dihedral, longitudinal dihedral, and diagonal tension or compression, respectively.

We vary each constant value independently by keeping the other two at their originally assumed values. Fig. 9 shows the results. No surprisingly, the 10-fold changes of 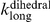 and 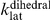 do not make notable differences on the energy and conformation evolutions. With changing 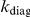, the energy barriers alternate. A smaller 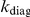-value results in a larger barrier, due to the increased flexibility of the model and the resultant larger tubulin displacements during the calculation process, but the stable state is soon found. Moreover, the equilibrium energy stepping remains the same and the energy barrier value is stable for each closure, so all conclusions in the paper can be validated.

#### Limitations and further directions

In order to elucidate a complete spatiotemporal evolution of conformation and energy during the microtubule growth, it is desired to authentically include the mechanisms of closure and hydrolysis in the model. This requires more explicit information about the details of these processes, e.g., direct experimental evidences for the evolutions of structure, interaction, and energy. Besides, the bond rupture, namely, the depolymerization is not considered in the presented model. We hope that a systematic modeling of the integrated dynamic process of tubulin assembly, sheet closure, and protofilament peeling can be accomplished in the next step.

## Supporting Information

Movie S1 **Sheet to tube transition process of a sheet-ended microtubule.** A sheet structure of ten monomers in length gradually experiences a curvature transition and closes into sheet.
(MOV)

Movie S2 **Growth simulation of a sheet-ended microtubule.** The tubulin assembly and sheet closure are in step with each other and the growth style is demonstrated alike Tetris.
(MOV)
